# Factors associated with health-related quality of life in people living with HIV in Norway

**DOI:** 10.1186/s12955-023-02098-x

**Published:** 2023-02-15

**Authors:** Vegard Skogen, Gudrun E. Rohde, Ranveig Langseth, Ole Rysstad, Tore Sørlie, Birgit Lie

**Affiliations:** 1 Department of Infectious Diseases, Division of Internal Medicine, University Hospital of North Norway, Tromsø, Norway; 2 Department of Clinical Medicine, Faculty of Health Sciences, University of Tromsø - The Arctic University of Norway, Breivika, 9037 Tromsø, Norway; 3 Department of Clinical Research, Sørlandet Hospital, Kristiansand, Norway; 4 Faculty of Health and Sport Sciences, University of Agder, Kristiansand, Norway; 5 Department of Internal Medicine, Sørlandet Hospital, Kristiansand, Norway; 6 Department of Mental Health and Substance Abuse, University Hospital of North Norway, Tromsø, Norway; 7 Department of Psychosomatic and Trauma, Sørlandet Hospital, Kristiansand, Norway

**Keywords:** HIV, Health-related quality of life, Short Form 36, Mental health, Somatic health

## Abstract

**Background:**

Despite the advances in the treatment of HIV, people living with HIV (PLHIV) still experience impairment of health-related quality of life (HRQOL). The aim of the study was to explore factors associated with HRQOL in a well-treated Norwegian HIV population.

**Methods:**

Two hundred and forty-five patients were recruited from two outpatient clinics to participate in this cross-sectional study of addiction, mental distress, post-traumatic stress disorder, fatigue, somatic health, and HRQOL. The latter was measured using the 36-Item Short Form Health Survey (SF-36). Stepwise multiple linear regression analysis was used to examine the adjusted associations between demographic and disease-related variables and HRQOL.

**Results:**

The study population was virologically and immunologically stable. Their mean age was 43.8 (SD = 11.7) years, 131 (54%) were men, and 33% were native Norwegians. Compared with the general population (published in previous studies), patients reported worse SF-36 scores for five of eight domains: mental health, general health, social function, physical role limitation, and emotional role limitation (all p < 0.001). Compared with men, women reported better SF-36 scores within the domains vitality (63.1 (23.6) vs. 55.9 (26.7), p = 0.026) and general health (73.4 (23.2) vs. 64.4 (30.1), p = 0.009). In the multivariate analyses, higher SF-36- physical component score values were independently associated with young age (p = 0.020), being employed, student, or pensioner (p = 0.009), low comorbidity score (p = 0.015), low anxiety and depression score (p = 0.015), being at risk of drug abuse (p = 0.037), and not being fatigued (p < 0.001). Higher SF-36-mental component score values were independently associated with older age (p = 0.018), being from a country outside Europe or from Norway (p = 0.029), shorter time since diagnosis, low anxiety and depression score (p < 0.001), answering ‘no’ regarding alcohol abuse (p = 0.013), and not being fatigued (p < 0.001).

**Conclusions:**

HRQOL was poorer in PLHIV than in the general population in Norway. It is important to focus on somatic and mental comorbidities when delivering health-care services in the ageing population of PLHIV to improve HRQOL even among a well-treated group of PLHIV as found in Norway.

## Introduction

HIV care has improved markedly over the years in locations with access to highly active antiretroviral treatment. HIV disease has changed from being a deadly infection to a chronic disease with a life expectancy approaching that of the general population [1]. Access to treatment is both life-saving for people living with HIV (PLHIV) and important from a public health perspective by preventing HIV transmission [2]. A change in focus for the treatment of ageing population of PLHIV requires knowledge of antiretroviral management as well as expertise in the prevention and management of comorbidities typically associated with ageing [3]. Despite the global awareness and improvement in HIV care, the extent to which the stigma and discrimination that remain adversely affect health-related quality of life (HRQOL) in (PLHIV) needs investigation.

The Joint United Nations Programme on HIV/AIDS launched its getting to zero vision as ‘zero new HIV-infections, zero HIV-related deaths, and zero HIV-related discrimination’ in 2011 [4]. Since then, the ‘90–90–90 treatment for all’ programme was established in 2017 to help end the AIDS epidemic by focusing on rapid diagnostics and adequate treatment [5]. The awareness and recognition of the importance of an integrated and people-centred health service of chronic care for PLHIV have led to a proposal by Lazarus et al. for a ‘fourth 90’ to focus on the quality of life (QOL) [6]. For people with chronic diseases who require lifelong treatment and care, QOL becomes a key point of care, and it is important to identify factors that influence QOL [7]. When focusing QOL in a health context, the concept HRQOL is often used.

It is generally accepted that HRQOL is a multidimensional concept that incorporates factors such as physical, cognitive, emotional, and social functioning, each of which can affect one’s disease and/or treatment [8]. Several assessment tools have been established to measure HRQOL among PLHIV [9]. Although various factors associated with HRQOL have been identified, there is no consensus about the main determinants within the socio-demographic, clinical, psychological, and behavioural factors [10]. Even well-treated PLHIV have reported poorer HRQOL compared with healthy controls [11], and PLHIV report poorer mental HRQOL than other with chronic diseases [12].

To our knowledge, identify factors associated with HRQOL among PLHIV residing in Norway have not been identified. The aim of this study was to explore the associations between HRQOL and gender, socio-demographic, mental, and somatic health variables in well-treated PLHIV residing in Norway.

## Material and methods

### Study population

All PLHIV older than 18 years who were registered at the HIV outpatient clinics at the Southern Hospital of Norway (SSHF) and University Hospital of North Norway (UNN) were eligible to participate in this cross-sectional study regardless of their language and literacy. The nurse-facilitated survey, *Mental health and quality of life among people living with HIV in Northern and Southern Norway,* was completed in October 2015 and included questionnaires containing 147 questions about socio-demographic background, fatigue, HRQOL, addiction, and somatic and mental health. Patients pre-diagnosed with a severe mental disorder or cognitive impairment that would make them incapable of answering the questions were excluded (n = 10). However, solely illicit drug use was not an exclusion criterion.

The study was approved by the Regional Committee for Medical Research Ethics (ref 2011/1925 REK Nord).

### Demographic and clinical data and questionnaires

The demographic data representing the independent variables included age, gender, hospital, education, cohabitation, and employment. The HIV-related variables were time since diagnosis, transmission route, openness about diagnosis, virus suppression, CD4+ cell count, antiretroviral therapy (ART), and treatment failure. The other health-related variables were, bodily pain, trouble sleeping, anaemia, and comorbidities (renal failure, thyroid disease, diabetes, cardiovascular disease, osteoporosis, arthritis, physical impairment, cancer, stroke, asthma, hepatitis C virus or chronic obstructive pulmonary disease). Comorbidity was defined as the presence of extra conditions beyond HIV. Data on medication, comorbidities, and blood test results were extracted from the medical records.

All participants completed seven validated instruments and a general informational scheme, conducted as a formalized interview with a trained nurse. The interviews were in English (n = 10), French (n = 2), or Norwegian (n = 224), or in another language with a professional interpreter if needed (n = 9). To explore anxiety and depression, the well-established Hopkins Symptom Checklist-25 (HSCL-25) was used [13]. This instrument has 10 items related to anxiety symptoms and 15 items to assess depression. The response options range from 1 to 4: ‘not at all’, ‘a little’, ‘quite a bit’, and ‘extremely’. The mean sum scores are calculated for the 10 anxiety items and for the 15 depression items, and a total score (average of all 25 items) is calculated. The HSCL-25 is a validated questionnaire that is useful as a screening tool in various settings, including in PLHIV [14].

To confirm depression, Beck’s Depression Inventory version 2 (BDI-II) was completed for participants with an HSCL-25 score above the cut-off of 1.75. BDI-II is a 21-question inventory designed to measure the severity of depression and comprises four statements for the time frame of two weeks. The answers are scored 0 to 3, and the responses are summed to yield a score that ranges from 0 to 63. A higher score indicates greater depression symptomatology, minimal depression (0–13), mild depression (14–19), moderate depression (20–28), severe depression (29–63) [15–17].

Diagnosing post-traumatic stress disorder (PTSD) can be challenging, and we used the Posttraumatic Stress Scale-16 (PTSS-16) as the screening instrument. The PTSS-16 comprises 16 questions about the frequency of symptoms after stressful life experiences during the past week. The answers are ‘not at all’, ‘a little bit’, ‘quite a bit’, and ‘almost always’, which are scored 1–4, respectively. A total mean score > 2.5 is defined as PTSD [18].

To explore risky alcohol consumption, the Alcohol Use Disorder Identification Test (AUDIT) was used. It is a widely used questionnaire of 10 items, each of which is scored as 0 to 4, and the higher score indicates greater alcohol consumption. We used the AUDIT score > 8 for men and > 6 for women to indicate risky consumption [19, 20]. The Drug Use Disorder Identification Test (DUDIT) is an 11-item questionnaire, each of which is scored as 0 to 4. Similar to the AUDIT, a higher score indicates at risk of drug abuse. DUDIT defines drugs as the misuse of legal drugs not prescribed by a doctor or the use of illicit drugs. The cut-off for at risk of drug abuse was a score of ≥ 6 for men and > 1 for women [21, 22].

The validated 11-item Chalder Fatigue Scale (FQ-11) contains two components, one to measure mental fatigue and the other to measure physical fatigue. Each item is scored on a 4-point Likert scale, and the total score is 0 to 11. Fatigue is defined as a score of ≥ 4 points [23–25].

HRQOL was assessed using the 36-item Short Form Health Survey questionnaire (SF-36), a self-reported and generic questionnaire that includes eight domains: general health, bodily pain, physical function, role limitations (physical), mental health, vitality, social function, and role limitations (emotional). The eight domains can be combined into a physical and mental sum scale that reflects physical and mental health. The physical component summary (SF-36-PCS) and the mental component summary (SF-36-MCS) scales were used in this study [26]. The SF-36 scales were scored according to published scoring procedures, and each scale was expressed using values from 0 to 100, with 100 representing excellent health [27–30].

### Statistical analyses

Statistical analyses were performed using IBM SPSS Statistics version 27 [31]. Continuous variables are presented as mean and standard deviation (SD), and categorical variables as numbers and percentages (%). The chi-squared test and Student’s *t* test were used to compare differences between subgroups. When comparing HRQOL of our study population with previous published data from the aged matched general Norwegian population [32, 33] we used GraphPad. In the GraphPad we included mean (SD) scores for the eight SF-36 domains from both populations, the number of participants and used Independent *t* test for comparison.

Stepwise multiple linear regression analysis (backward procedure) was used to examine the adjusted associations between demographic and disease-related variables and HRQOL (SF-36-PCS and SF-36-MCS scores) (PIN = 0.05 and POUT = 0.20). Assumptions for linear regression were checked and fulfilled. The independent variables in the multiple analyses were chosen based on univariate associations with HRQOL and clinical experience/relevance and included age, gender, cohabitation, native continent, employment status, comorbidities, HIV viral load, at risk of drug or alcohol abuse, and fatigue scores [34]. The final tested variables are listed in Table 3. For robustness, we also tested the models using forward multiple regression analyses. The level of significance was set at p < 0.05.

## Results

### Demographic and disease-related characteristics

The SSHF had 121 registered PLHIV and the UNN 158. Of the total of 279 PLHIV, 245 completed the survey, giving a response rate of 87.8%. The mean age of the participants was 43.8 (SD = 11.7) years; 131 (54%) were men and 33% were native Norwegians. Close to 60% had < 13 years of education, and 30% were either unemployed, undergoing rehabilitation, or disabled. The time since the diagnosis of HIV was a mean 9.4 (SD = 7.4) years; 86% had a viral load < 50 copies/mL, and their average CD4+ count was 0.53 × 10^9^/L (SD = 0.26). Fifty-three (22%) of the participants were not open about their HIV status to their closest family or partner. There was a significant difference between PLHIV in northern and southern Norway (p = 0.01), however there were no significant gender differences. Thirty-five (14%) of the study population were open about their HIV status in the public, i.e. at work. Though, there were no significant differences among gender or hospital. The socio-demographic characteristics of the cohort are presented in Table 1, which shows the similarities and differences between men and women and hospital in age, native country, employment status, educational level, cohabitation status, and fatigue levels.

**Table 1:** Demographic and clinical variables among people living with HIV in Southern (n = 109) and Northern (n = 136) Norway (n = 245)

		Gender			Hospital		
	All n = 245	Women n = 114	Men n = 131	p-value	UNN n = 136	SSHF n = 109	p-value
**Demographic variables**							
Age, (y) mean (SD)	43.8 (11.7)	41.7 (10.1)	45.6 (12.3)	.008*	44.3 (11.9)	43.0 (11.5)	.420
*Cohabitation, n (%)*							.037*
Living together	108 (44%)	60 (53%)	48 (37%)	.012*	68 (50%)	40 (37%)	
Living alone	137 (56%)	54 (47%)	83 (63%)		68 (50%)	69 (63%)	
*Education, n (%)*				< .001*			.042*
< 13 years	142 (58%)	80 (70%)	62 (47%)		71 (52%)	71 (65%)	
≥ 13 years	103 (42%)	34 (30%)	69 (53%)		65 (48%)	38 (35%)	
*Employment status*^*1*^*, n (%)*				.757			.072
Employed/student/pensioner	169 (70%)	79 (71%)	90 (69%)		101 (74%)	68 (64%)	
Unemployed/rehabilitation/ disabled	74 (30%)	33 (29%)	41 (31%)		35 (26%)	39 (36%)	
*Native country/continent, n (%)*				< .001*			.950
Norway	81 (33%)	16 (14%)	65 (49%)		47 (35%)	34 (31%)	
Rest of Europe	15 (6%)	5 (4%)	10 (7%)		8 (6%)	8 (7%)	
Americas	10 (4%)	1 (1%)	9 (7%)		5 (4%)	5 (5%)	
Asia	39 (16%)	30 (26%)	9 (7%)		22 (16%)	16 (15%)	
Africa	100 (41%)	62 (54%)	38 (30%)		54 (40%)	46 (42%)	
*Citizenship*^*2*^*, n (%)*				< .001*			.731
Asylum seeker	8 (3%)	6 (5%)	2 (2%)		4 (3%)	4 (4%)	
Refugee with residence permit	50 (21%)	26 (23%)	24 (18%)		30 (22%)	20 (18%)	
Non-Western immigrant	45 (18%)	32 (28%)	13 (10%)		27 (20%)	18 (17%)	
Western immigrant	8 (3%)	3 (3%)	5 (4%)		3 (2%)	5 (5%)	
Norwegian Citizen	133 (55%)	46 (41%)	87 (66%)		72 (53%)	61 (56%)	
Not informed closest relatives about HIV	53 (22%)	23 (18%)	30 (26%)	.097	19 (14%)	34 (31%)	.001*
Open about HIV	35 (14%)	23 (18%)	12 (11%)	.117	24 (18%)	11 (10%)	.093
**Clinical variables**							
Time since diagnosis, (y) mean (SD)	9.4 (7.4)	9.5 (7.1)	9.3 (7.7)	.764	9.9	8.7	.195
*Transmission route of HIV, n (%)*				< .001*			.553
Heterosexual	98 (40%)	53 (56%)	45 (34%)		63 (46%)	35 (32%)	
Homosexual/MSM	31 (13%)	0	31 (24%)		18 (13%)	13 (12%)	
Needle sharing/blood transfusion/perinatal	9 (4%)	3 (3%)	6 (5%)		5 (4%)	4 (4%)	
Unknown	107 (43%)	39 (41%)	49 (37%)		50 (37%)	57 (52%)	
Antiretroviral therapy, n (%)	229 (94%)	105 (92%)	124 (95%)	.429	128 (94%)	101 (93%)	.646
HIV viral load < 50 (copies/mL), n (%)	212 (86%)	98 (86%)	114 (87%)	.809	119 (88%)	93 (85%)	.620
CD4+ (× 10^9^/L), mean (SD)	0.53 (0.26)	0.52 (0.22)	0.53 (0.29)	.566	0.53 (0.28)	0.53 (0.25)	.816
HSCL-25, mean (SD)	1.64 (0.58)	1.58 (0.48)	1.69 (0.65)	.156	1.57 (0.6)	1.74 (0.59)	.020*
Mental distress, at risk, n (%)	78 (32%)	31 (27%)	47 (40%)	.146	36 (27%)	42 (39%)	.044*
BDI-II^3^, mean (SD)	22.9 (14.1)	19.4 (9.8)	22.3 (12.3)	.277	21.9 (12.8)	23.8 (15.1)	.596
*BDI-II*^*3*^*, n (%)*							
Minimal depression	16 (7%)	8 (7%)	8 (6%)		8 (6%)	8 (7%)	
Mild depression	25 (10%)	8 (7%)	17 (13%)		14 (10%)	11 (10%)	
Moderate depression	15 (6%)	8 (7%)	7 (5%)		5 (4%)	10 (9%)	
Severe depression	20 (8%)	6 (5%)	14 (11%)		9 (7%)	11 (10%)	
Post-traumatic stress disorder^4^, n (%)	16 (7%)	4 (4%)	12 (9%)	.077	8 (6%)	8 (7%)	.633
Drug abuse, at risk n (%)	18 (7%)	4 (4%)	14 (11%)	.032*	10 (7%)	9 (8%)	.793
Alcohol abuse, at risk, n (%)	36 (15%)	2 (2%)	34 (26%)	< .001*	16 (12%)	20 (18%)	.148
Fatigued, n (%)	94 (38%)	38 (33%)	56 (43%)	.191	46 (34%)	48 (44%)	.102
Comorbidity, mean (SD)	0.4 (0.8)	0.3 (0.7)	0.5 (0.8)	.020*	0.6 (0.9)	0.2 (0.5)	< .001*

*UNN* University Hospital of North Norway, *SSHF* Southern Hospital of Norway

^1^Employment status, n = 243, 2 missing

^2^Citizenship, n = 244, 1 missing, MSM: men who have sex with men, CD4+ : T lymphocytes bearing the CD4 + receptor, HSCL-25: Hopkins Symptoms Checklist-25, Mental distress, at risk: HSCL-25 > 1.75 (range 1–4)

^3^BDI-II: Beck Depression Inventory version 2 (range 0–63), minimal depression (0–13), mild depression (14–19), moderate depression (20–28), severe depression (29–63), performed on mental distress, at risk, n = 76, percentage shown for total n = 243, 2 missing

^4^Post-traumatic stress disorder: Post Traumatic Stress Scale-16 > 2,5 (range 0–4), n = 244, 1 missing, Drug abuse, at risk: Drug Use Disorder Identification Test > 4 (range 0–44), Alcohol abuse, at risk: Alcohol Use Disorder Identification Test > 8 (range 0–40), Fatigued: Chalder Fatigue Scale > 4 (range 0–11), Comorbidity: comorbidity (range 0–3). Continuous variables are presented as the mean and standard deviation (SD) and categorical variables as number and percentage (%). Chi-squared (categorical variables) tests and Student’s *t* tests (continuous variables) were used to compare differences between groups

*Significant at 5% level

### HRQOL in PLHIV

Comparison of PLHIV treated at the two hospitals showed that those treated at UNN had better scores for two SF-36 domains: mental health (76.2 (20.4) vs. 70.1 (23.7), p = 0.033) and social function (82.6 (27.7) vs. 74.8 (30.1), p = 0.037). PLHIV treated at the UNN also had a higher SF-36-MCS (48.0 (13.3) vs. 43.6 (14.1), p = 0.015). The PLHIV at both hospitals were part of the study, so in further analyses the patients were considered as one group despite some small differences (data not shown).

Compared with men, women reported better SF-36 scores within the domains vitality (63.1 (23.6) vs. 55.9 (26.7), p = 0.026) and general health (73.4 (23.2) vs. 64.4 (30.1), p = 0.009) (Table 2). Comparison between PLHIV and with data from an age matched general Norwegian population [32, 33] showed that PLHIV had worse SF-36 scores for five of the eight domains: mental health, general health, social function, physical role limitations, and emotional role limitations (all p < 0.001) (data not shown).

**Table 2:** Health-related quality of life in people living with HIV using the 36-item Short Form Health Survey questionnaire (n = 245)

	All n = 245	Women n = 114	Men n = 131	p-value
*Health-related quality of life*				
SF-36, mean (SD)				
Mental health	73.5 (22.1)	73.0 (21.0)	73.9 (23.1)	.731
Vitality	59.3 (25.5)	63.1 (23.6)	55.9 (26.7)	.026*
Bodily pain	70.7 (29.6)	71.1 (27.1)	70.4 (31.8)	.850
General health	68.6 (27.4)	73.4 (23.2)	64.4 (30.1)	.009*
Social function	79.2 (29.0)	82.7 (26.1)	76.1 (31.2)	.072
Physical function	84.9 (20.6)	86.4 (17.8)	83.6 (22.8)	.287
Physical role limitations	67.5 (40.9)	72.2 (39.3)	63.4 (42.0)	.092
Emotional role limitations	74.4 (39.1)	76.0 (38.0)	73.0 (40.1)	.549
SF-36-PCS	49.3 (10.2)	50.6 (8.7)	48.8 (11.3)	.058
SF-36-MCS	46.0 (13.8)	46.7 (12.6)	45.9 (14.7)	.511

*SF-36* 36-item Short Form Health Survey questionnaire, *SF-36-PCS* physical component summary, *SF-36-MCS* mental component summary, *HRQOL* Health-related quality of life. Data are shown as the mean and standard deviation (SD). The SF-36 range is 0–100, where 100 indicates a high HRQOL. Student’s independent-sample *t* tests were used for comparisons between groups

*Significant at 5% level

### Adjusted associations between demographic and clinical variables and HRQOL

In the multivariate analyses (Table 3), lower SF-36-PCS values were independently associated with old age (B = -0.12 (95% CI [− 0.21; − 0.02], p = 0.020), being unemployed/undergoing rehabilitation/disabled (B = − 6.79 (95% CI [− 6.00; − 0.98)], p = 0.007), higher comorbidity score (B = − 2.46 (95% CI [− 4.04 to 0.88)], p = 0.015), higher HSCL-25 score (B = − 3.18 (95% CI [− 5.73; − 0.62)], p = 0.015), and being fatigued (B = − 6.79 (95% CI [− 9.62; − 3.96)], p < 0.001), while higher SF-36 PCS values was associated with being at risk of drug abuse (B = 3.14 (95% CI [0.20;6.09)], p = 0.037). Lower SF-36-MCS values were independently associated with being from Europe except from Norway (B = − 5.14 (95% CI [− 9.75; − 0.54], p = 0.029), longer time since diagnosis (B = − 0.16 (95% CI [− 0.32;0.00)], p = 0.046), higher HSCL-25 score (B = − 15.01 (95% CI [− 17.49; − 12.53]), p < 0.001), being at risk of alcohol abuse (B = − 4.14 (95% CI [− 7.41; − 0.88)], p = 0.013), and being fatigued (B = − 5.49 (95% CI [− 8.36; − 2.61], (p < 0.001), while higher SF-36 MCS values was associated with lower age (B = 0.12 (95% CI [0.02;0.23)], p = 0.018). The demographic and clinical variables included in the full model explained 34.2% of the variance for the SF-36-PCS, in the final model 35.2%. The independent variables in the full model explained 61.9 of the variance for the SF-26-MCS, in the final model 62.9%. The same results were seen when the multivariate models were run forwards and if including hospital in the model (data not shown).

**Table 3:** Stepwise multivariate regression model of the adjusted associations between demographic and clinical variables, and physical and mental components of Health-related quality of life in people living with HIV (n = 245)

	SF-36-PCS				SF-36-MCS			
	Full model B (95% CI)	p-value	Final model B (95% CI)	p-value	Full model B (95% CI)	p-value	Final model B (95% CI)	p-value
**Demographic variables**								
Age (y)	–0.11 (–0.23, –0.00)	.045*	–0.12 (–0.21, –0.02)	.020*	0.14 (0.02, 0.25)	.020*	0.12 (0.02, 0.23)	.018*
Woman	2.16 (–0.35, 4.66)	.091	1.76 (–0.58, 4.11)	.140	–2.82 (–5.38, –0.26)	.031*	–2.08 (–4.42, 0.27)	.083
Living together	0.45 (–1.84, 2.74)	.669			–0.25 (–2.59, 2.10)	.837		
*Native continent*								
Norway	0.88 (–2.25, 4.00)	.581			–1.82 (–5.02, 1.38)	.264		
Rest of Europe	1.44 (–3.34, 6.23)	.553			–5.86 (–10.76, –0.96)	.019*	–5.14 (–9.75, –0.54)	.029*
America	3.95 (–1.73, 9.63)	.172			–2.44 (–8.25, 3.37)	.409		
Asia Africa (ref)	–2.33 (–5.62, 0.96)	.164	–2.57 (–5.59, 0.45)	.094	0.99 (–2.37, 4.36)	.561		
Unemployed/undergoing rehabilitation/disabled	–3.40 (–5.95, –0.84)	.009*	–6.79 (–6.00, –0.98)	.007*	1.45 (–1.16, 4.07)	.275		
**Clinical variables**								
Comorbidity	–2.50 (–4.13, –0.87)	.003*	–2.46 (–4.04, –0.88)	.015*	0.19 (–1.48, 1.86)	.824		
HIV viral load < 50 copies m/L	0.88 (–2.26, 4.01)	.583			–1.48 (–4.69, 1.73)	.365		
Time since diagnosis (y)	–0.02 (–0.18, 0.14)	.826			–0.15 (–0.31, 0.02)	.080	–0.16 (–0.32, –0.00)	.046*
HSCL-25	–3.31 (–5.97, –0.65)	.015*	–3.18 (–5.73, –0.62)	.015*	–15.31 (–18.04, –12.59)	< .001*	–15.01 (–17.49, –12.53)	< .001*
Drug abuse, at risk	3.12 (–1.32, 5.37)	.233	3.14 (0.20, 6.09)	.037*	–2.39 (–5.50, 0.72)	.131	–2.43 (–5.44, 0.59)	.114
Alcohol abuse, at risk	2.03 (–1.32, 5.37)	.233	2.28 (–0.93, 5.49)	.162	–3.60 (–7.03, –0.18)	.039*	–4.14 (–7.41, –0.88)	.013*
Fatigued	–6.75 (–9.66, –3.84)	< .001*	–6.79 (–9.62, –3.96)	< .001*	–5.47 (–8.46, –2.49)	< .001*	–5.49 (–8.36, –2.61)	< .001*
**R**^**2**^	34.2%		35.2%		61.9%		62.4%	

The final model used a backward-step procedure to define the included variables. The full model included all of the selected variables entered into the model, and the final model included variables in the final step using the backward procedure

*CI* confidence interval, *SF-36* 36-item Short Form Health Survey questionnaire (range 0–100) where 100 indicates a high Health-related quality of life, *SF-36-PCS* physical component summary (range 0–100), *SF-36-MCS* mental component summary (range 0–100), Comorbidity: comorbidity including Hepatitis C (range 0–10), Hopkins Symptoms Checklist-25 (range 1–4), Drug abuse, at risk: Drug Use Disorder Identification Test > 1(f) or > 6 (m) (range 0–44), Alcohol abuse, at risk: Alcohol Use Disorder Identification Test > 6 (f) or > 8 (m) (range 0–40), Fatigued: Chalder Fatigue Scale > 4 (range 0–11)

*Significant at 5% level

## Discussion

In this cross-sectional survey of 245 well-treated PLHIV residing in Norway, PLHIV had a poorer HRQOL than the general population [32, 33]. This observation is consistent with the results of other recently published cross-sectional surveys of HRQOL in PLHIV [7, 11].

The study population was recruited from two hospitals and differed significantly on two HRQOL domains (mental health and social function) as well as the SF-36-MCS. This finding was surprising for two reasons. First, all residents in Norway have access to free, high-quality health-care services and other social support systems [35]. Second, the SSHF has established a user-driven HIV clinic within their hospital facilities to provide optimal holistic health care and treatment, and to empower PLHIV [36]. The differences in HRQOL seen in the two hospitals in our study may reflect the fact that 42% of those living in northern Norway had full-time work compared with only 27% of those living in southern Norway. An association between employment and HRQOL on both the SF-36-PCS and SF-36-MCS has been reported in several studies [37–42]. A recent study among Swedish adults, showed that unemployment is strongly related to a poorer HRQOL [43]. However, unemployment hits groups of individuals differently and should be considered when prioritizing labour market measures [34]. The study is of interest, especially in a Norwegian setting, due to our common Scandinavian welfare and social model. In addition, the population in southern Norway more often lived alone, had a lower educational level, less open regarding the HIV to closest relatives or partner, and were more often disabled pensioners compared with the population living in northern Norway. These factors may influence HRQOL, as previously reported by Degroote and colleagues [10].

We explored whether age was associated with higher SF-36-MCS and SF-36-PCS scores. Several studies have reported that old age is associated with lower physical health scores [41, 44–49]; which may indicate poorer physical functioning and more comorbidities because of older age. Like previous studies that have reported a positive correlation between increasing age and better mental health, we found that older PLHIV reported better mental health and MCS scores [40, 47]. However, the literature is inconsistent regarding the relationship between age and mental health [50–52].

Another demographic variable associated with mental health was the native continent of the PLHIV in this study; that is, coming from a country outside Europe or from Norway was associated with a higher score at SF-36-MCS. A considerable number of our European study population were from Eastern Europe or the former Soviet Union, which may have contributed to their low SF-36-MCS-scores. Studies from this region have reported the need to focus on health and social care to improve HRQOL and QOL [39, 53–55]. A recent publication by Kuznetsov and colleagues, focused on treatment and health challenges among PLHIV residing in the Russian Federation [56], where especially HIV stigma makes a great challenge [57]. Our findings might be a result of previous experiences of living with HIV, despite the fact that they are now living in Norway.

Along with age, comorbidity was another variable that was significantly associated with lower SF-36-PCS scores in our study population. We defined comorbidity as the sum of somatic conditions, but not including mental distress, addiction, or fatigue, which were independent variables in our multivariate regression model. However, a significant association between somatic comorbidity and SF-36-MCS was not seen. Our comorbidity results are consistent with the results of several other studies [12, 58–60]. The population of PLHIV, is an ageing population with comorbidities [61, 62], and this must be addressed in the clinical setting to improve HRQOL in PLHIV.

In our study, low mental distress measured by the HSCL-25 was strongly associated with high SF-36-PCS and SF-36-MCS scores. Studies reported in the review by Degroote and colleagues show that depression and anxiety have a negative impact on HRQOL [10]. Another significant factor associated with HRQOL in our study was addiction, as measured with the DUDIT and AUDIT. Increased alcohol intake was significantly associated with lower SF-36-MCS, which suggests that alcohol consumption is associated with impaired mental HRQOL. By contrast, drug use was associated with improved physical HRQOL. This finding was not expected, and may reflect the exclusion of intravenous drug users with cognitive and mental disorders from this study. Few studies have focused on HRQOL and substance abuse in PLHIV [10]. In our study, fatigue was also associated with poorer mental and physical HRQOL, an observation that has been reported previously [63, 64]. Taken together, previous research and our findings highlight the importance of focusing on comorbidity/multimorbidity in the treatment of PLHIV.

## Strengths and limitations

The strengths of our study are the high response rate, the fact that few participants were excluded, and that no variables were missing from the regression analyses. Further recruitment through scheduled clinical follow-ups and data collection by trained nurses likely increased the data accuracy as compared with data obtained from self-referral and self-report. The inclusion of PLHIV in Norway who do not speak the national language and people with poor reading and writing skills likely helped to improve the study’s external validity. Another strength of our study is that our study population is approximately 8% of the PLHIV residing in Norway. The northern and the southern counties in Norway have PLHIV from urban and rural settings with a similar socio-demographic characteristic as PLHIV in Norway [65].

The study’s cross-sectional design means that causality cannot be established. Another limitation is the number of variables that could be entered into the final regression model because we had 245 participants and, in turn, some estimates had wide confidence intervals. To limit the possible effects of confounding variables, all variables identified previously as confounders and independent variables were adjusted in the final regression model.

It might also be considered as a weakness that some of the questions included in the different PROMS, e.g. HSCL-25 and the questions included in the SF-36 MCS score, could be considered to be quite similar. However, to measure the concepts in focus of our study (e.g. anxiety/depression and the mental part of HRQOL) we choose to keep the original, well validated, questionnaires to do so. Another limitation in our study, is the fact that we did not include a validated questionnaire to measure stigma and discrimination in relation to living with HIV. However, we included two questions regarding openness.

## Implications and future research

In the comparison between the PLHIV and the general population, patients reported worse scores for five of eight domains of the SF-36. The findings of this study contribute to knowledge about how age, employment status, somatic and psychological comorbidities, addiction, and fatigue are associated with HRQOL among PLHIV in a developed country. The study population was a well-treated population of PLHIV residing in Norway. Our findings emphasize the importance of focusing on comorbidities in the ageing PLHIV to optimize their HRQOL. Further studies using a longitudinal design are needed to increase the knowledge of HRQOL among PLHIV in the global setting.

## Conclusion

We found poorer HRQOL among PLHIV in Norway than in the general population. HRQOL was influenced by several concurrent variables associated with poorer mental and physical HRQOL. It is important to focus on somatic and mental comorbidities in the delivery of health-care services for the ageing population of PLHIV to improve QOL, even among the virologically and immunologically stable group of PLHIV as in Norway.

## Data Availability

The datasets used and/or analysed during the current study are not publicly available because of the General Data Protection Regulation laws of Norway but are available from the corresponding author on reasonable request and with permission from the Norwegian Centre for Research Data.
